# Mechanochemical Approaches to Fundamental Studies in Soft‐Matter Physics

**DOI:** 10.1002/anie.202402442

**Published:** 2024-03-15

**Authors:** Robert T. O'Neill, Roman Boulatov

**Affiliations:** ^1^ Department of Chemistry University of Liverpool University of Liverpool Department of Chemistry Grove Street Liverpool L69 7ZD

**Keywords:** mechanochemistry, polymers, allostery, soft-matter, macromolecular rheology

## Abstract

Stretching a segment of a polymer beyond its contour length makes its (primarily backbone) bonds more dissociatively labile, which enables polymer mechanochemistry. Integrating some backbone bonds into suitably designed molecular moieties yields mechanistically and kinetically diverse chemistry, which is becoming increasingly exploitable. Examples include, most prominently, attempts to improve mechanical properties of bulk polymers, as well as prospective applications in drug delivery and synthesis. This review aims to highlight an emerging effort to apply the concepts and experimental tools of mechanochemistry to fundamental physical questions in soft matter. A succinct summary of the state‐of‐the‐knowledge of the field, with emphasis on foundational concepts and generalizable observations, is followed by analysis of 3 recent examples of mechanochemistry yielding molecular‐level details of elastomer failure, macromolecular chain dynamics in elongational flows and kinetic allostery. We conclude with reasons to assume that the highlighted approaches are generalizable to a broader range of physical problems than considered to date.

## Graphical abstract

The promise of polymer mechanochemistry to improve mechanical properties of polymers is widely recognized. This review highlights a complementary, so‐far underexploited, potential of this emerging field to yield quantitative molecular insights into many complex physical problems in soft matter, of both practical and fundamental significance, including fractures of mechanically‐loaded materials and dynamics of macromolecules in rapid flows.

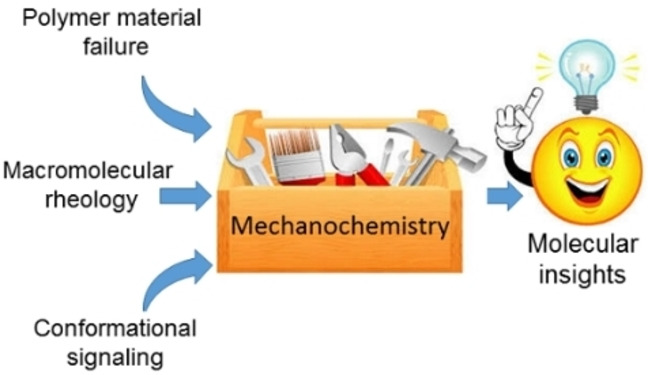

## 1 Background on State‐of‐the‐Knowledge

### 1.1 Key Phenomenology

Polymer mechanochemistry aims at discovering, understanding and exploiting chemical reactions promoted by stretching polymer segments beyond their strain‐free contour lengths.^[1]^ Such usually‐transient overstretching results from diverse physical processes, both biological and technological, in polymer solutions,^[2]^ melts, powders,^[3]^ molecular networks,^[4]^ and at interfaces.^[5]^ Additionally, micromanipulation techniques allow exquisitely controlled stretching of individual polymer chains,^[6]^ albeit at a cost of spectroscopic product characterization.

**Figure 1:**
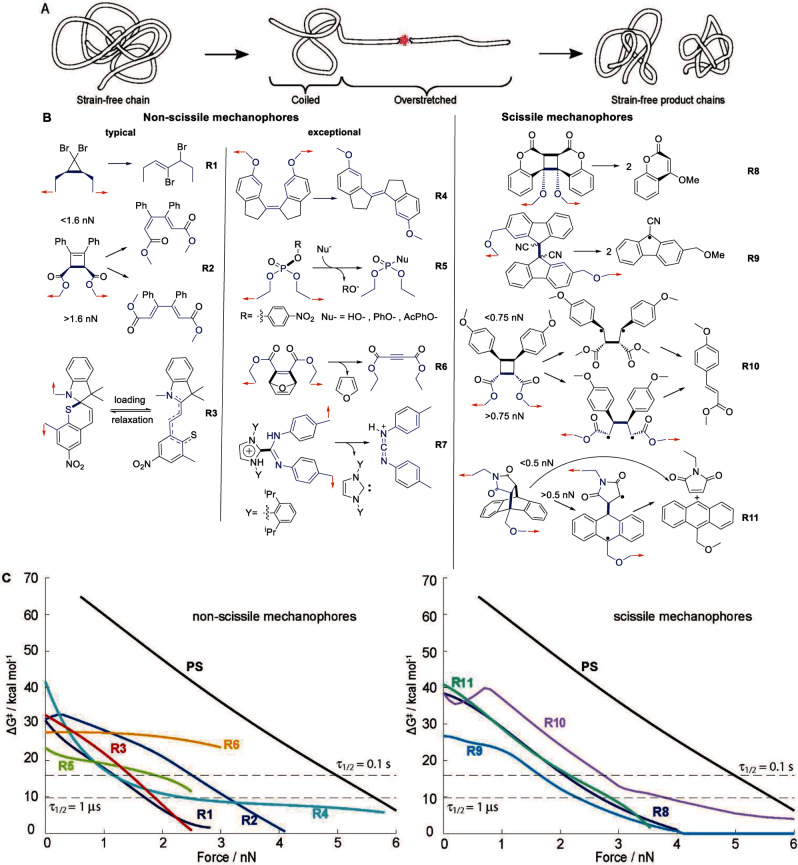
Illustrative mechanochemical reactions. (A) Mechanochemical fracture of an overstretched backbone in a generic linear polymer. (B) Examples of non‐scissile and scissile mechanophores: the polymer‐backbone contributing bonds are blue; the scissile bonds bold; red arrows show the pulling axis. Rare mechanochemically dethreadable rotaxanes[^29^, ^30^] constitute a separate type of scissile mechanophores excluded here because they have been insufficiently studied. (C) Reported force‐dependent activation free energies, ΔG^≠^. Homolysis of the backbone C−C bond in polystyrene in black; the dashed horizontal lines correspond to maximum ΔG^≠^ observable in sonicated solutions (9.8 kcal/mol) or in single‐molecule force experiments (15.6 kcal/mol). In R5‐R6, competing homolysis of a P−O or C−O backbone mechanophore bond outcompetes dissociation of the non‐backbone bond (bold) at 2.6 and 3.1 nN, respectively, limiting the achievable mechanochemical acceleration. Force dependences of kinetic barriers are far more diverse than assumed either by the popular Bell‐Evans model^[31]^ or the COGEF method. For multibarrier reactions or competing reaction mechanisms, apparent ΔG^≠^ for mechanophore consumption is plotted, calculated from the reported individual barriers according to^[21]^ and,^[8]^ respectively. Highly non‐monotonic ΔG^≠^(*f*) of R10 at <1 nN reflects sequential change in the rate‐determining TS, followed by change in the minimum‐energy reaction path, with increasing force: refs. [^8^, ^32^] report the other examples. Table S1 lists the sources and DFT model chemistries of all plotted ΔG^≠^.

Most reactions in polymer mechanochemistry start with dissociations of a backbone bond^[7]^ (for the few exceptions,[^8^, ^9^, ^10^] see Figure 1). Longest‐known, mechanistically simplest and practically most significant is homolysis of a single covalent bond, usually C−C, C−O, C−S and S−S,^[2]^ which contribute to gradual degradation (or aging) of most, maybe all, commercial polymers under large or repeated mechanical loads.^[11]^

**Figure 2:**
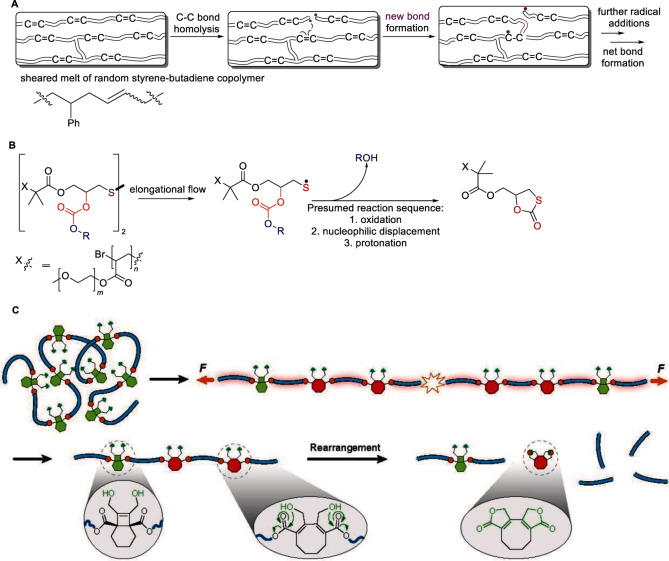
Examples of reaction cascades wherein the strain‐free products of the initial mechanochemical backbone bond dissociation react spontaneously in the absence of force to form new backbone bonds (A),^[11]^ release a small molecule, ROH (B)^[33]^ or depolymerize (C, adapted from^[34]^). Non‐selective chain fracture in B is omitted for space.

Numerous reactive moieties contain one or more covalent bonds that are dissociatively more labile, usually over a range of stretching forces achievable by at least one practical loading scenario, than the backbones of common polymers such as polyacrylates or polystyrene (PS, Figure 1c). This allows mechanochemistry of such moieties under tensile load to be studied by attaching a suitably‐long polymer chain to each of two atoms of the moiety and overstretching a portion of the resulting backbone (Figure 1b). The trade name for small molecules that are inert under ambient temperature but isomerize or fragment when part of an overstretched backbone is mechanophore. Some mechanophores generate different products mechanochemically and when heated (e.g., R2, Figure 1b), and others yield the same products but by different mechanisms (R10–R11). Products of many known mechanochemical reactions are isolable but some react spontaneously, either reverting to the (strain‐free) reactant (R3, Figure 1) or initiating a reaction cascade (Figure 2). Very few yield new isolable mechanophores,^[12]^ reflecting the difficulty of creating sequential mechanochemical reactions with desired combinations of activation barriers at experimentally accessible tensile loads.^[7]^

**Figure 3:**
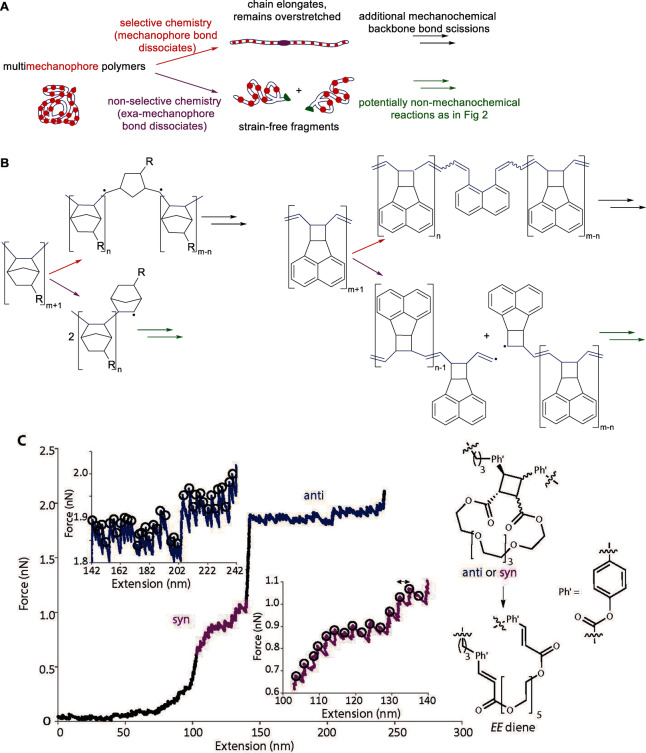
Multimechanophore polymers. (A) The distinct outcomes of selective and non‐selective mechanochemistry in such polymers. (B) Examples of polymers of highest possible linear density of mechanophores and the primary products of their selective and non‐selective chemistries. The backbone bonds are in blue: note the change in the mechanophore bonds contributing to the backbone upon mechanophore isomerization. (C) Evidence of sequential reactions of individual non‐scissile mechanophores in a polymer of macrocyclic cinnamate dimers. The highlighted portions of the force/extension curve correspond to dissociation of the cyclobutane core in the two isomers of the dimers (reaction on the right). Circled force maxima correspond to dissociation of the 2^nd^ scissile bond of each cyclobutane. The sawtooth pattern results from inertial mechanical reequilibration of the AFM and the overstretched polymer chain following contour‐length extension by cyclobutane dissociation. Panel C is adapted from.^[13]^

If the dissociating bond of an overstretched backbone is outside a mechanophore, the chain is said to fracture non‐selectively (Figure 3), otherwise, either a selective chain fracture results (scissile mechanophore) or the number of backbone bonds and hence the chain contour length increases (a non‐scissile mechanophore). A scissile mechanophore can be made non‐scissile by incorporating it into a macrocycle.[^13^, ^14^, ^15^, ^16^] Outside a few careful single‐molecule force (SMF) experiments,^[13]^ dissociation of backbone bonds within and beyond mechanophores compete, i.e., 100 % selective polymer mechanochemistry is inaccessible in bulk.

Tensile load can also accelerate the formation of new covalent bonds, either directly or indirectly. The former has only a few examples, including concerted scission/formation of bonds in mechanochemical displacements,[^8^, ^17^, ^18^, ^19^, ^20^, ^21^, ^22^] a reductive elimination example,^[23]^ and additions of a small molecule to a (presumably) overstretched backbone.[^12^, ^24^] Some of these reactions are accompanied by backbone fracture but none increases the number of backbone bonds.^[7]^ Conversely, chain fracture is the obligatory precursor for indirect bond formation, because it generates either a highly reactive macroradical or a catalyst that initiates spontaneous formation of new load‐bearing bonds.^[25]^ Compositions of some widely used engineering elastomers may reflect empirical optimization of such bond‐forming cascades well before their existence was recognized.^[11]^

**Figure 4:**
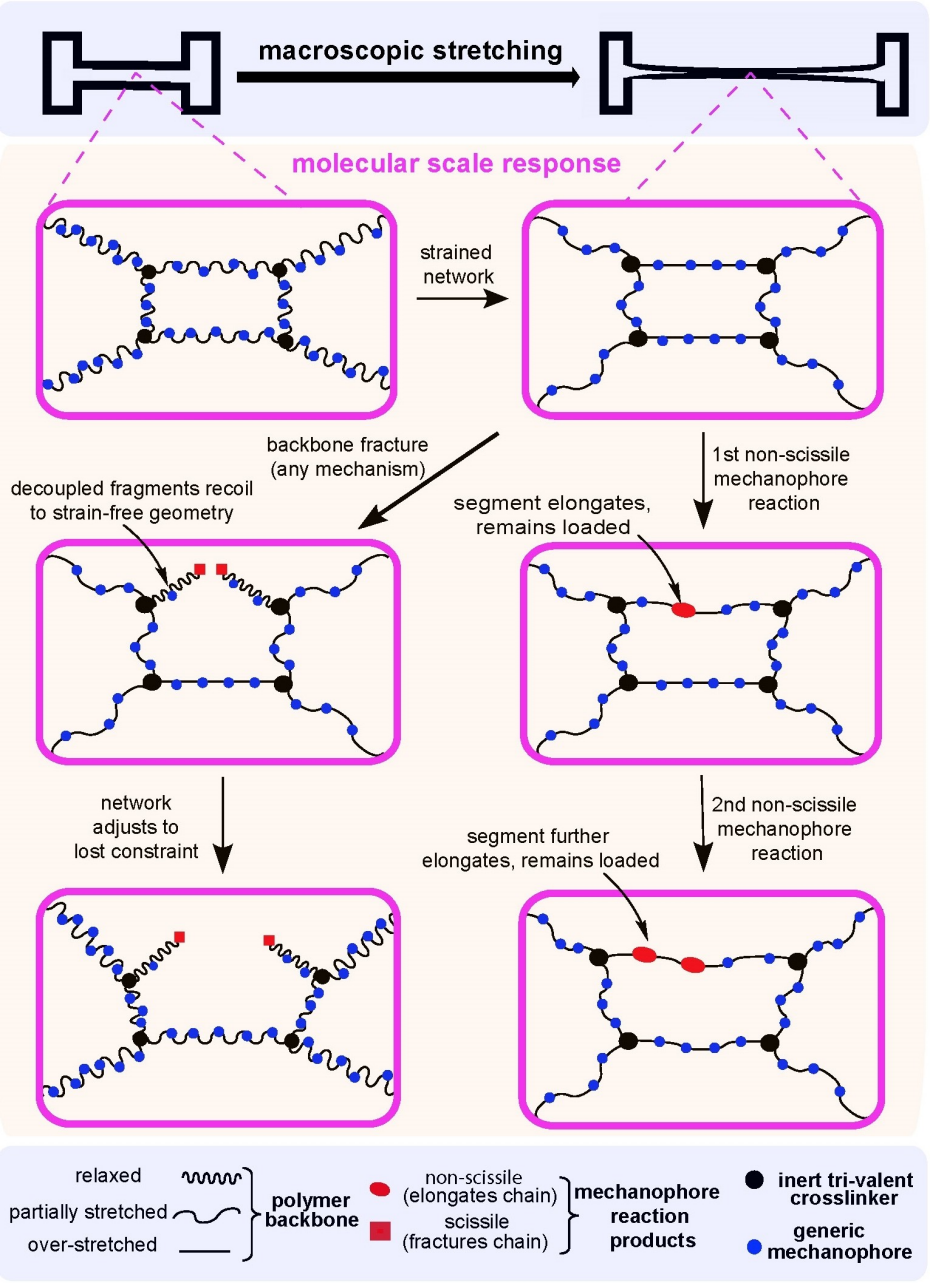
Molecular mechanism that supports sequential non‐scissile mechanochemical reactions but limits chain fracture to a single site in an elastomer.

In an overstretched chain containing multiple non‐scissile mechanophores, the backbone bond of each mechanophore dissociates sequentially,[^13^, ^26^] as demonstrated by single‐molecule force measurements (Figure 3), sometimes doubling the strain‐free contour lengths.[^27^, ^28^] In contrast, any overstretched chain fractures at a single site regardless of how many scissile bonds it has. While backbone bond dissociation partially relaxes the backbone strain, at least transiently, only backbone fracture instantaneously dissipates it, yielding a pair of shorter, strain‐free, product chains. The difference reflects the distinct physical mechanisms that maintains the overstretched backbone long enough for it to react mechanochemically (Figure 4).

In the simplest scenario, a chain segment between two crosslinks in a mechanically loaded elastomer is overstretched when the strained macroscopic geometry requires the crosslinks to separate beyond the segment contour length. When one of its non‐scissile mechanophores reacts, the segment elongates, momentarily reducing its pulling force on the crosslinks. In response, the local molecular network reequilibrates mechanically, restoring the tensile load on the segment (close) to that prior to the reaction and causing another mechanophore to react, repeating the cycle. The same process causes the characteristic sawtooth pattern in force/extension curves of non‐scissile multimechanophore chains in single‐molecule force experiments (Figure 3c). Conversely, backbone fracture uncouples the segment geometry from the separation of the two crosslinks, allowing the product fragments to recoil (presumably very rapidly) to strain‐free and thus mechanochemically‐inert geometries.

In rapidly flowing polymer solutions and in sheared polymer melts overstretching results not from the geometric constraints imposed by the macroscopic dimensions of the material, but a dynamic competition between conformational entropy, which favors a coiled chain, and the friction between the chain and the flow (of either the solvent or other chains), which reduces when the backbone aligns with the flow direction. The probability of a backbone segment to become overstretched increases steeply with the backbone length and the fluid strain rate, which quantifies how much the flow accelerates along the flow direction. A fluid strain rate sufficient to reduce the half‐life of a backbone bond to the sub‐μs timescale (approximate persistence of overstretched geometries in practically achievable flows) is too slow to overstretch the shorter chains produced by backbone fracture. Consequently, any kinetically‐significant strain stored in the backbone traversing the scissile transition state, probably dissipates on the timescale of vibrational energy redistribution, followed by much slower conformational transitions to coiled chains. Only if and when such chains encounter much greater fluid strain rates than those required to generate them can they react mechanochemically. In contrast, bond dissociation within a non‐scissile mechanophore elongates the chain, which increases its viscous friction, the tensile load on it by the flow and thus its propensity to react both selectively and non‐selectively.

**Figure 5:**
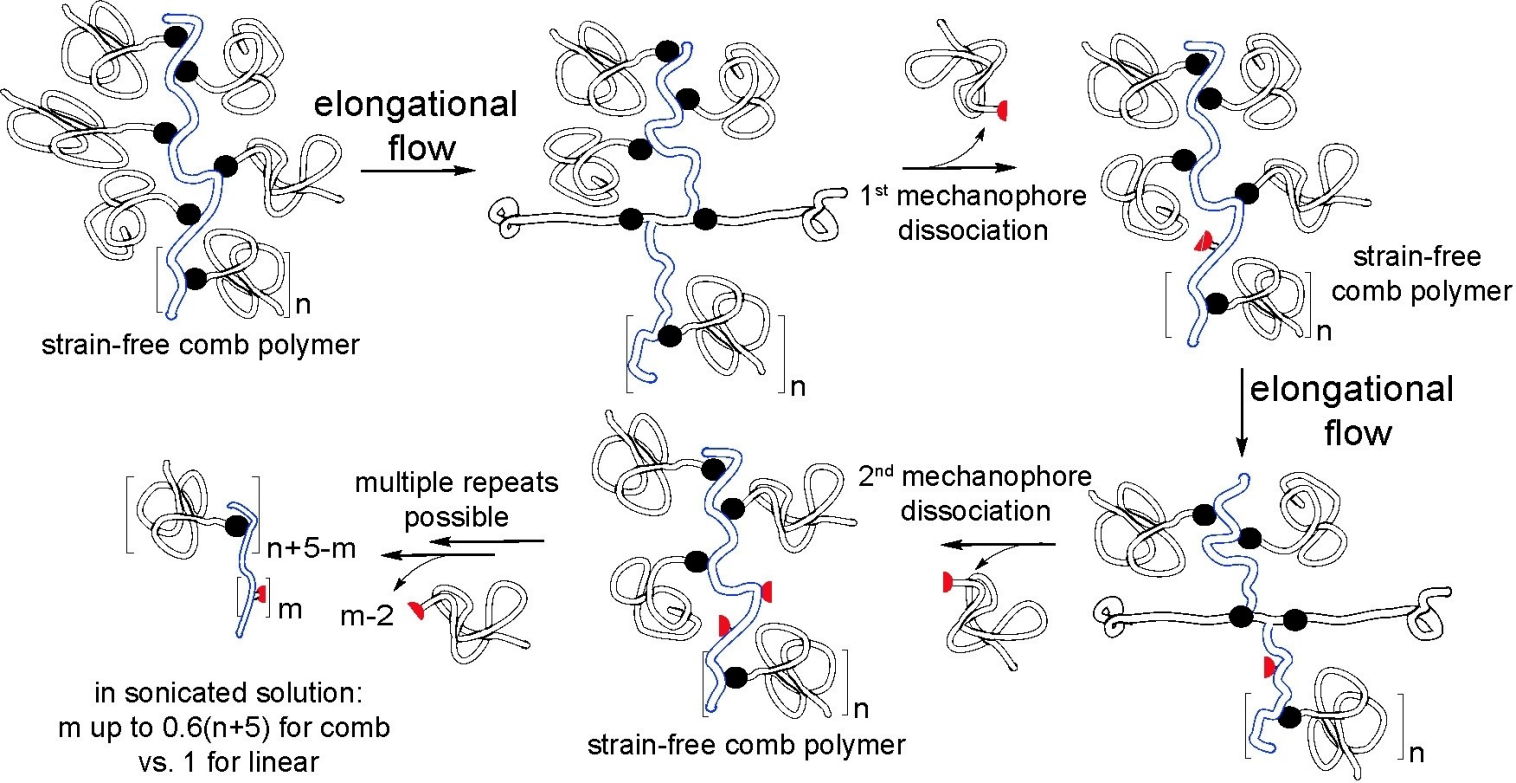
Molecular mechanism enabling sequential scissile mechanochemistry in a comb polymer.

The self‐limiting nature of mechanochemical chain fracture favors non‐scissile multimechanophore polymers for most practical applications because such polymers likely change their composition more, and thus generate a greater mechanochemical response, than dissociatively labile backbones under comparable mechanical loading. Non‐linear chains, such as comb polymers containing scissile mechanophores in each side‐chain, mitigate, but not eliminate, the low per‐chain yield of scissile mechanochemistry (Figure 5).^[35]^

### 1.2 Physicochemical Foundations

Stretching a polymer backbone accelerates a reaction if its rate‐determining transition state, rdTS, causes partial relaxation of bond lengths, angles and torsions elsewhere in the polymer, whose distortions reflect the overstretched geometry of the backbone. This partial relaxation requires the reactive moiety to elongate along the stretching axis as it traverses its rdTS. Qualitatively the same mechanism of molecular strain relief accelerates reactions in polymer mechanochemistry and in small strained organic molecules but the magnitude of the effect, and a means of quantifying it are distinct. Because a polymer backbone is much longer than any mechanophore, even a sub‐Å elongation of the latter in its rdTS can reduce the strain energy of the rest of the backbone by tens of kcal/mol. This explains how stretching polystyrene lowers the activation barrier of homolysis of its C−C bond by 75 kcal/mol, whereas strain‐induced barrier lowering of >15 kcal/mol is exceptionally rare in small organic molecules.^[36]^

**Figure 6:**
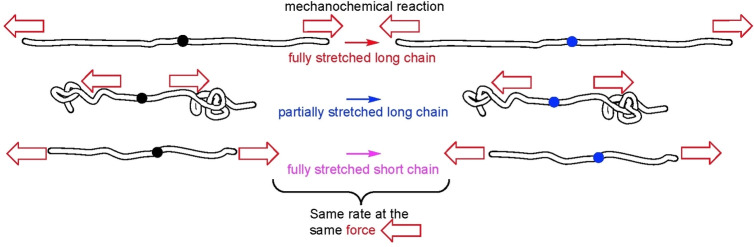
In an internally mechanically‐equilibrated overstretched backbone, the stretching force acting across any pair of backbone atoms defines the local force responsible for the reactivity of an embedded mechanophore. Consequently, this reactivity can be discussed quantitatively without considering the rest of the polymer.

In polymer mechanochemistry this kinetically‐significant molecular strain is quantified not in terms of strain energy, but force, because the latter is intensive (scale‐independent). A single mechanophore in an internally‐mechanically equilibrated backbone stretched with the same force across any pair of backbone atoms at either side of the mechanophore has the same time‐dependent reaction probability regardless of the chain length (Figure 6). This allows even quantitative discussions of localized reactivity in overstretched chain (segments) to dispense with the macromolecule and focus on the much smaller, more tractable reactive site, whose kinetic and thermodynamic stabilities as a function of force are amenable to quantum‐chemical calculations.^[37]^ Although no systematic effort to estimate the accuracy of such calculations has appeared, several measured single‐molecule force/extension curves[^13^, ^15^, ^16^, ^38^] and product distributions observed in some sonication experiments[^12^, ^16^] were reproduced accurately with activation energies calculated at the DFT‐level.

A long‐standing effort in polymer mechanochemistry is to learn how to control mechanochemical kinetics through molecular design. Highly force‐sensitive reactions are more likely to (a) outcompete non‐selective chain fractures over the broad distribution of local stretching forces that are thought to control mechanochemistry in bulk and (b) maximize differential stabilities in the absence of load and under practical loading scenarios. Both attributes are essential for practical applications.

**Figure 7:**
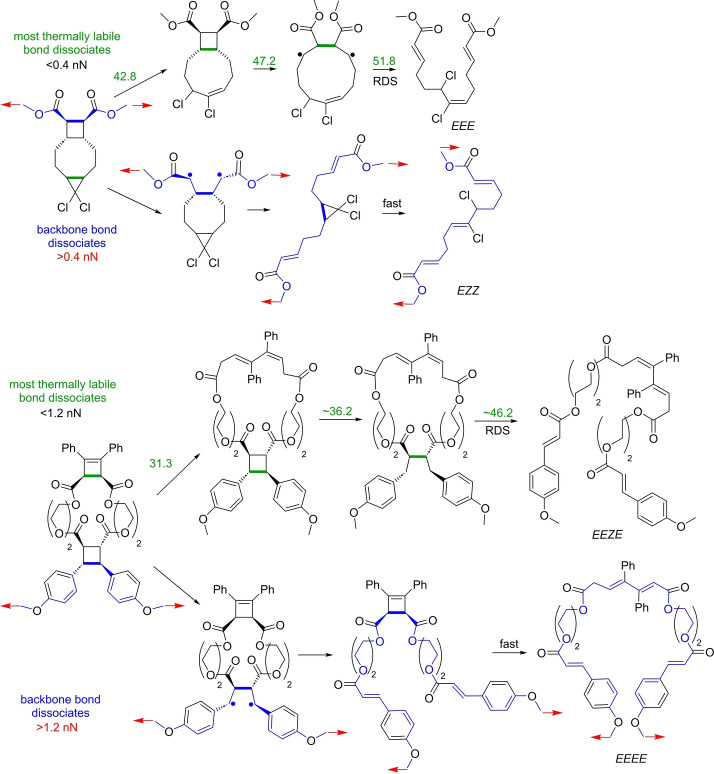
Mechanochemical gating exploits the capacity of backbone overstretching to change which bond dissociates preferentially. High force overrides the sequence of bond dissociations determined by the relative heights of strain‐free barriers (green numbers in kcal/mol) by making only the scissile bond that comprises the backbone (blue bonds) in each intermediate kinetically competent to dissociate. Note the different products at low and high force. Red arrows indicate the external force vectors.

The most general structure/reactivity relationship of polymer mechanochemistry to date is that stretching a backbone only labilizes backbone bonds (R5–R8, Figure 1b, are the rare exceptions of accelerated scission of a side bond of a backbone atom). A putative mechanophore whose scissile bond(s) is outside the backbone is unaffected by how much the backbone is stretched. In mechanophores with multiple scissile bonds the order in which these bonds dissociate in a stretched polymer is controllable by the choice of the atoms through which the mechanophore connects to the remaining backbone. This enables mechanochemical gating, which prevents dissociation of a labile scissile bond until more inert scissile bonds dissociate by sequestering the former in a molecular loop excluded from the backbone until the backbone‐contributing inert bonds dissociate (Figure 7). The propensity of overstretching to labilize only backbone bonds also turns concerted dissociations of strain‐free Diels–Alder adducts stepwise with a biradical intermediate under stretching force (R10–R11, Figure 1b). Precluding this mechanistic switch makes the reaction force‐insensitive.^[32]^

Since the sequence of bonds defining a backbone is not always unique, an objective, quantitative analysis of the effect of molecular structure, including the location of the chain/mechanophore attachment atoms, on the mechanochemical kinetics relies on restoring forces of individual bonds.^[39]^ These are calculated from the stretching force in harmonic oscillator approximation using technically‐straightforward^[40]^ and theoretically validated^[39]^ approaches. The restoring force of the scissile bond across mechanistically diverse reactions correlates with their measured or computed dissociation kinetics much better than the backbone stretching force.^[8]^

The choice of the mechanophore atoms connecting it to the backbone is the most studied molecular design parameter to adjust the threshold force at which the mechanophore reacts at a desired rate,[^41^, ^42^, ^43^] or the resulting product distribution.^[12]^ In sonicated solutions the placement of the mechanophore relative to the chain center of mass,^[44]^ the primary (homopolymer vs. block copolymers[^44^, ^45^]), secondary (linear, cyclic,^[46]^ brush^[35]^), or strain‐free tertiary (random coil, intramolecularly crosslinked coil,^[47]^ helix^[48]^) chain structure affect the kinetics of mechanophore‐centered chemistry, mechanochemical selectivity (the number of reacted mechanophores per chain fracture) or both. The kinetics of non‐selective chain fracture in sonicated solutions, melts and milled powders is also sensitive to chain topology (across a range of linear, cyclic, star, dendronized, brush and randomly branched topologies).[^3^, ^11^, ^49^]

## 2 Molecular Events Enabling Elastomer Failure

Despite the enormous technological, economic and scientific importance of elastomers, and the concomitant volume of studies, molecular events responsible for elastomer failure remain poorly understood, complicated by the difficulties of correlating mechanical properties of a bulk polymer with its molecular structure and composition, and of experimentally validating, benchmarking and parameterizing molecular models.

A popular approach exploits mechanochromic reactions, whose reactant and product(s) have distinguishable absorption and/or emission characteristics.[^50^, ^51^, ^52^] Observed mechanochromic responses, which are typically changes in the emission or absorption of a volume of the loaded material integrated over time period Δt, are relatable to the total number of mechanochromic reactions that occurred during Δt. Consequently, scissile mechanochromes allow estimates of, for example, the density of fractured polymer strands as a function of the distance from a propagating tear in an axially stretched elastomer, thereby enabling experimental differentiation among distinct hypotheses of how stressed polymer networks behave at mesoscale.^[53]^

Conversely, the local force that a reacting mechanochrome experiences and how it evolves during Δt is not yet inferable from measured mechanochromic response without some drastic assumptions. The fundamental reason is that the probability of a mechanochemical reaction to occur increases both with force acting on the reacting moiety and the time it acts and this reaction probability alone cannot be deconvoluted into the individual components.^[7]^ For example, the same fraction of anthracene/maleimide adducts (R11, Figure 1) dissociate when subjected to 2 nN for 0.1 s, or 3 nN for 0.1 μs. In a mechanically loaded molecular network the range of local forces and times consistent with the observed change in optical properties is much larger still because the microscopic conditions that determine the kinetic stability of each mechanochrome (such as loading rates and persistence times) vary both over Δt and across the monitored volume of the loaded material.^[4]^

Constraining these ranges requires the accurate force‐dependent activation free energy of the mechanochromic reaction, ΔG^≠^(*f*) and of any other competing reactions (e.g., non‐selective chain fracture or another mechanochromic reaction). DFT‐level calculations validated against single‐molecule force measurements^[13]^ or model studies^[55]^ yield reliable ΔG^≠^(*f*) estimates, whereas aphysical methods, such as COGEF are unlikely to produce even qualitatively credible interpretations. Semi‐quantitative correlations of mechanochromic responses across related materials have been exploited productively without the knowledge of underlying microkinetics.[^56^, ^57^] Finally, time‐dependent emission or absorption intensity of a reversible mechanochrome reflects its “instantaneous” local load, which simplifies quantitative interpretation of the response. However, detectable intensity changes are limited to forces corresponding to the equilibrium constant between >0.1 and <10. All reported to date reversible mechanochromic reactions[^51^, ^58^] meet this criterion only at <100 pN, because these reactions exploit low‐barrier conformational transitions, which are eliminated by forces far below the fracture limit of the network.^[59]^ Even spiropyran/merocyanines isomerization (e.g., R3, Figure 1), which becomes detectable at <350 pN, is irreversible in bulk materials at relevant timescales.^[51]^

Whereas a mechanochromic response cannot yet be transformed into temporaspatial distributions of local forces across a mechanically loaded network, neither conceptual, nor unsurmountable technical barriers appear to preclude the inverse of estimating the mechanochromic response from such distributions. As a result, mechanochromism allows quantitative experimental tests of suitably detailed molecular models of strained elastomers. The next section illustrates an application of this capacity for macromolecules in transient flows. Simultaneously, underutilized alternative experimental approaches are better suited to quantifying the molecular response to bulk polymers to mechanical load.

**Figure 8:**
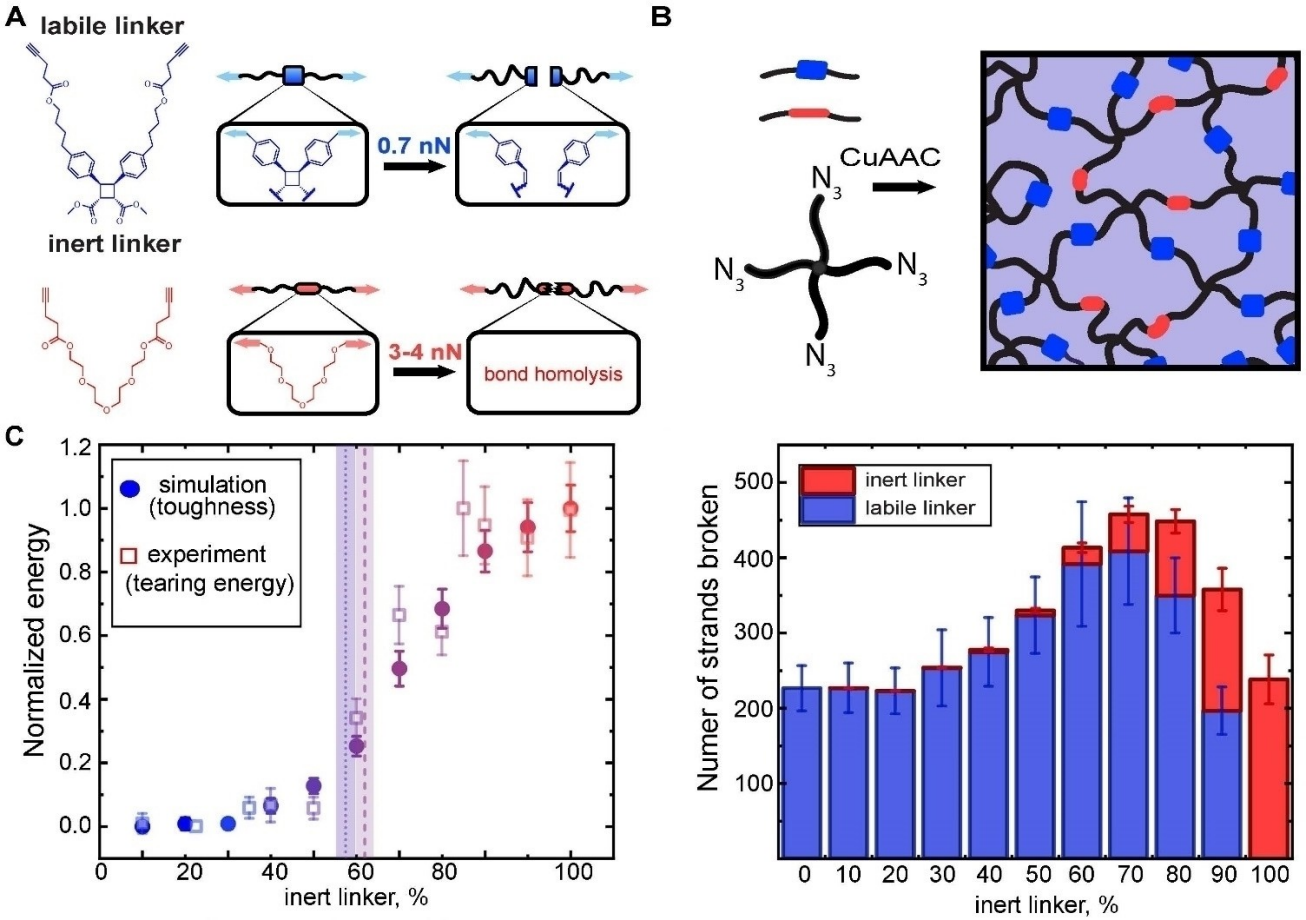
Mechanochemical control of crack propagation. (A) The studied networks were formed by linking 4‐arm PEGs with either dissociatively labile or inert linkers. (B) The sigmoidal dependence of the tearing energy on the linker composition supports the role of local network depercolation in crack propagation. The vertical lines defined estimated depercolation thresholds. (C) The number of labile linkers that break before simulated material fails does not decrease when some are swapped for inert ones even when depercolation requires fracture of some inert linkers. Adapted from.^[54]^

One such alternative is to correlate failure characteristics of a material to mechanochemical kinetics of scissile mechanophores uniformly distributed within its molecular network. The limited kinetic diversity of known mechanochromic reactions make this approach practical only with non‐mechanochromes. Its recent application^[54]^ tested the role of local network depercolation—fracture of enough strands to turn a part of the network into a mix of isolated fragments—in crack propagation. It measured the tearing energy of a series of close‐to‐uniform networks of 4‐arm star PEG oligomers connected by either only dissociatively labile linkers, only inert linkers, or a mixture of the two. The former was scissile cinnamate dimer whose mechanochemical kinetics was previously quantified experimentally and computationally,^[13]^ and the latter triethylene glycol whose fracture behavior was assumed to resemble that of the rest of the backbones. All networks had depercolation threshold (the minimum fraction of strands whose fracture solubilizes the gel) of ~0.5, yet the measured tearing energy was independent of the fraction of the inert linkers below 0.4, or above 0.7 and rose steeply ~10‐fold at intermediate compositions (Figure 8). No known molecular model of fracture predicts this dependence.

Coarse‐grained simulations revealed that replacing an increasing fraction of labile strands with inert ones didn't reduce, and even increased, the number of dissociated labile strands at fracture. This inverse correlation of the probability of labile strand failure and its fraction in the network suggests that a subset of labile strand failures is incidental, i.e., not contributing to crack‐enabling network depercolation. This conclusion is consistent with both increasing delocalization of failed labile strands with increasing fraction of the inert linkers and the increasing number of such dissociations occurring at material strains exceeding the failure strain of the all‐labile network. The latter results from the inert strand having a lower fracture probability than the labile analog of the same contour length at any macroscopic strain.

The results are plausibly generalizable to networks of chemically‐identical strands of varying lengths, where shorter strands act as labile links but the distribution of incidental strand failure across the material and the role of internal mechanical disequilibria in this distribution remain to be understood. Incidental strand failure may further complicate molecular interpretations of spatially distributed mechanochromism, exacerbated by the lower threshold force of such reactions compared to dissociation of other backbone bonds, their presence in only some strands, and the variations in strand lengths. A recently demonstrated^[60]^ secondary mechanochromism may offer a partial workaround.

## 3 Macromolecular Chain Dynamics in Transient Elongational Flows

Dilute solutions of polymers have diverse uses in everyday life, research and industry. Unlike small‐molecule solutes, macromolecules are very sensitive to how and how fast the solution flows, responding to such flows in complex ways, from undergoing transient and fully reversible conformational transitions which change the tertiary structure of the solute (e.g., uncoiling) at low flow rates to flow‐induced covalent mechanochemistry, including chain fracture, at high fluid strain rates. Gaining detailed molecular descriptions of such behavior has been prevented by the lack of experimental tools to determine the molecular chain geometries and the dynamics of their evolution in practically‐relevant flows.

High temporospatial heterogeneity of such flows precludes direct structural characterization of chains in them, which contrasts with the extensively‐studied slow steady flows where individual ultralong DNA solutes are optically tractable. In mechanochemistry‐inducing flows, the changing composition of a flowing solution reflects the convolution of the probabilities of individual chains to partition among microenvironments with distinct flow field characteristics (e.g., strain rates and persistence time) and the relative probabilities of each backbone bond of a chain in each such subpopulation to react. Because the latter depends on the chain geometry within a time period preceding the reaction, the changing composition of the flowing solution contains information about backbone structures leading to the reaction. When 2 or more competing mechanochemical reactions with experimentally distinguishable force‐dependent kinetics control the composition, chain geometries are inferable without the knowledge of the size of individual subpopulations, which cannot yet be estimated experimentally or computationally.

We recently demonstrated the potential of this approach by characterizing non‐equilibrium macromolecular geometries in dilute sonicated solutions.^[61]^ Solution ultrasonication has multiple industrial and laboratory applications and is the most common experimental tool of overstretching polymer backbones. Sonication causes cavitation, the continuous formation and violent collapse of numerous microscopic gas bubbles. A sub‐μm thick layer of liquid around an imploding cavitation bubble is accelerated to strain rates potentially exceeding 10^10^ s^−1^, which overstretches even short macromolecules in these layers and causes them to react mechanochemically. Diverse physical processes beyond acoustic energy input cause cavitation and affect macromolecular solutes similarly. The short duration of such flows (<10 μs) and the tiny volumes comprising them at any time make characterization of cavitationally‐induced chain dynamics particularly challenging.

**Figure 9:**
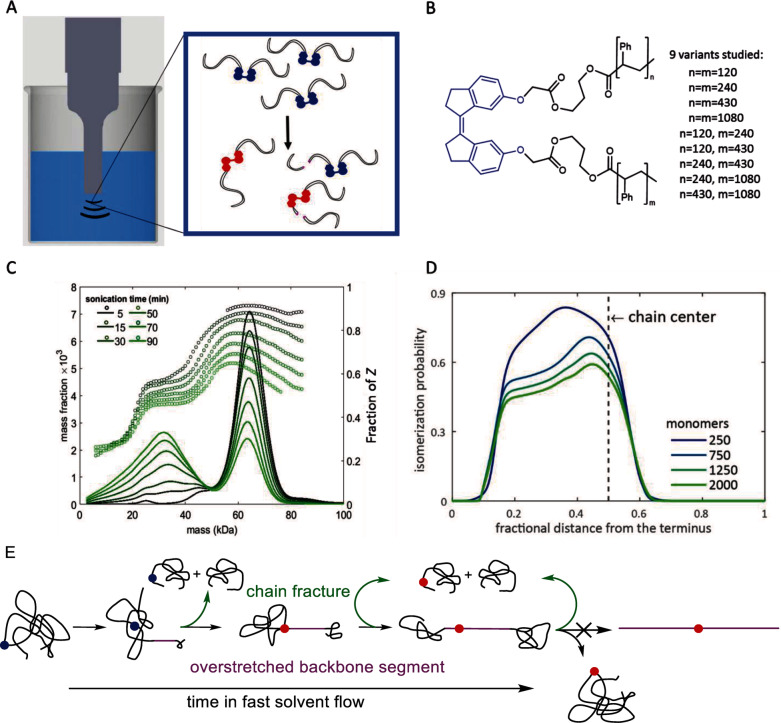
Mechanochemistry reveals chain dynamics in rapid solvent flows. (A–B) Ultrasonication of a dilute solution of polystyrenes containing a single *Z* stiff stilbene (blue structure in A and blue moiety in B) per chain creates competition between mechanochemical chain fracture and *Z*→*E* isomerization (red structures in A) both within and between chains. (C) Chain fracture changes the initial uniform mass distribution of the polymer solute to bimodal (solid lines); isomerization reduces the fractions of *Z* isomer among stiff‐stilbene‐containing chains and makes it vary strongly with chain mass (dotted lines). (D) Modelling this data revealed how force experienced by each backbone bond and its propensity to react mechanochemically vary along a backbone. Here, the variation of the total probability of *Z*‐SS isomerization along the chain is shown. (E) Various distributions combine into the picture of chain response in the flow dominated by competition between structural and bond rearrangements, with chains never becoming fully overstretched. Panels C–D are adapted from.^[61]^

We quantified the dynamics of linear polystyrenes containing single *Z*‐stiff stilbene per backbone (R4, Figure 1b). Overstretching such a chain accelerates both chain fracture by homolysis of any C−C backbone bond and *Z*→*E* isomerization of its lone stiff stilbene (Figure 9). Mechanochromism of the latter enables simple quantitation of the fraction of *Z* and *E* stiff stilbenes in chains of every length and thus of the relative probabilities of each reaction to occur in the same macromolecule (to yield a short *E*‐containing chain), or in separate chains (to yield either a short *Z*‐containing fragment or a long *E*‐containing chain). The ratios of these probabilities directly inform on the temporal evolution of the number and relative locations of the backbone bonds experiencing force between 2.5 and 5.8 nN. By measuring a series of polystyrenes spanning a 12‐fold range of contour lengths and containing stiff stilbene at varying locations, we mapped how the force of each backbone bond evolves in chains of 50–600 nm long in accelerating solvent flows. These force maps upend several key assumptions that have traditionally informed the design and interpretation of experiments in polymer mechanochemistry.

At all contour lengths, the overstretched portion of the backbone is both short (<30 %) and drifts rapidly (e.g., on the order of 0.1 m/s at fracture) towards the center, which exposes many more backbone bonds to sufficient force to react mechanochemically on the timescale of a bubble collapse than a conventional model of macromolecule‐as‐a‐rigid‐rod allows (Figure 9D). This drift explains the otherwise‐puzzling previous observations of mechanochemical reactivity far from centers of linear chains[^16^, ^44^, ^62^] and suggest hitherto‐unrecognized opportunities to tune mechanochemical reactivity in transient flows.

Chains remain partially overstretched for 0.6–2.5 μs before either fracturing or the flow dissipating. The physical mechanism that maintains a backbone in internal mechanical disequilibrium for >1 μs is unknown. First, 1 μs of flow at the minimum strain rate of >10^9^ s^−1^ needed to affect the geometry of a flexible chain such as PS with contour length <0.5 μm in a low‐viscosity solvent such as THF, corresponds to accumulated (Hencky) strain at least 10x that thought to be sufficient to fully uncoil the chain.^[63]^ Second, internal equilibration of mechanochemically reacting chains is unlikely to require slow thermally activated conformational transitions responsible for the persistent conformational heterogeneity of macromolecular solutes in flows that are too slow to cause mechanochemistry. Instead, very fast intramolecular vibrational energy flows (IVR) are the most plausible mechanism by which the enthalpic molecular strain responsible for mechanochemistry at >2 nN redistributes within a macromolecule.^[59]^ Both arguments seemly preclude the existence of internal mechanical disequilibria in individual backbones on μs‐timescales. These considerations suggest to us a gap in the current understanding of intra‐ and intermolecular energy flows in highly non‐equilibrium environments.

## 4 Quantitative Molecular Analysis of Kinetic Allostery

The simplest allosteric molecule has three functional components: a receptor site, a substrate site and molecular scaffold connecting the two. Binding of the ligand (effector) to the receptor site changes either the kinetic stability of the substrate site or its affinity for another molecule. Detailed molecular mechanisms by which the two sites couple across the intervening molecular scaffold are critical for understanding diverse biochemical processes and for designing sophisticated reaction networks and feedback loops capable of information processing and signaling, and that may help answer questions as fundamental as the origin of life. Yet despite extensive empirical data on allosteric biopolymers and significant effort to develop synthetic allosteric analogues, a general, quantifiable and predictive atomistic description of such mechanisms remain to be developed.

**Figure 10:**
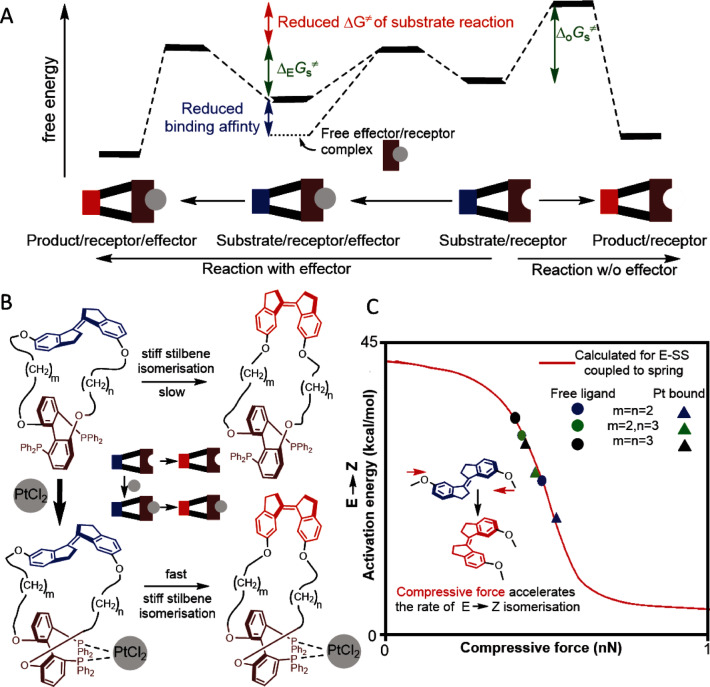
A mechanochemical analysis of allostery. (A) Energy diagrams illustrating the simplest example of kinetic allostery. The effector (grey) reduces the reaction barrier of a substrate (blue) coupled to a receptor (brown), Δ_E_G_s_^≠^, relative to the same reaction without the effector, Δ_O_G_s_^≠^, at the expense of the lower affinity of the receptor for the effector (blue arrow). The ratio of the blue‐to‐red arrows defines allosteric efficiency. (B) The series of macrocycles containing *E*‐stiff stilbene (blue) as substrate coupled to the diphosphine receptor (brown) used for validating the mechanochemical model of kinetic allostery. (C) The substrate reaction—isomerisation of *E* to *Z* stilbene—is accelerated up to 10^4^ fold by the binding of PtCl_2_ (effector). Computed force‐dependent ΔG^≠^ of isomerization (red line) reproduces accurately the measured kinetics across the series (symbols). Panels B and C are adapted from.^[64]^

The long‐range coupling that yields allostery has conceptual parallels with the conversion of directional mechanical motion to kinetically‐significant molecular strain responsible for polymer mechanochemistry.^[1]^ Consequently, the force formalism of mechanochemical kinetics may offer a general approach to analyzing quantitatively how the properties of the molecular scaffold determine flow of structural information between allosteric sites. This approach was applied to allosteric acceleration of reaction kinetics (Figure 10a), the least understood form of allostery that has proven largely impossible to replicate synthetically.

A minimal models of kinetic allostery was created with a series of bifunctional macrocycles,^[64]^ each containing *E* stiff stilbene as the substrate whose *E*→*Z* isomerization was accelerated by coordination of Pt^2+^ (effector) to a biphenyl biphosphine receptor at the opposite site of the macrocycle (Figure 10b). Across the 4 macrocycles of increasing sizes, Pt binding accelerated *E*→*Z* isomerization by up to 10^4^‐fold. The measured variation of the activation free energy of isomerization, ΔG^≠^, across both Pt‐free and Pt‐bound macrocycles was reproduced quantitatively by representing macrocycles beyond the substrate as harmonic springs of the same compliance but with the equilibrium distances that decrease with both macrocycle size and upon Pt binding. Consequently, the observed allosteric accelerations reflect a single continuous correlation between force on the substrate from the rest of the molecule and substrate ΔG^≠^ (Figure 10c), despite the variation in the composition of the macrocycles. The result is consistent with the previous findings that restoring force is useful in isolating the contribution of molecular strain to observed kinetics from any other rate‐determining factors even in non‐macromolecular substrates.

Reducing the kinetic effects of both the effector binding and the molecular scaffold across which the substrate and receptor sites communicate to a single physical parameter reveals a broad and likely generalizable relationship between the molecular structure and the efficiency of allosteric coupling, defined as the ratio of the effector‐induced barrier lowering to the reduction in the affinity of the receptor for the effector (Figure 10a). This efficiency places the upper limit on the practically achievable magnitude of allosteric acceleration. It depends non‐monotonically on a combination of scaffold stiffness, the difference in compliances of the substrate moiety in the reactant and transition states, and the substrate strain prior to effector binding as quantified by its restoring force. Appreciable accelerations require a narrow and non‐intuitive combination of these parameters, which may explain the previous lack of synthetic examples of kinetic allostery. It also predicts a seemingly‐aphysical above‐unit efficiency of moderate accelerations achievable in pre‐strained allosteric reactants. The conclusions from this model remain to be validated in other allosteric systems, including polymers. The established capacity of force to quantify the distribution and kinetic effects of both entropic and enthalpic molecular strain,^[19]^ regardless of the molecular size,^[38]^ suggests that both the approach and its specific predictions are not limited to a particular molecular architecture, composition or reaction mechanism.

## 5 Summary and Outlook

A broad consensus is emerging that polymer mechanochemistry has the potential to improve the mechanical properties of diverse polymers to practically‐interesting extents.[^4^, ^14^, ^65^] Discovery of mechanochemically enhanced polymers is one of the two dominant thrusts of contemporary polymer mechanochemistry (the other being search for new mechanochemical reactivity^[1]^). Commercially‐impactful empirical effort at exploiting macroradicals from mechanochemical chain fracture to improve mechanical properties of polymers during synthesis, processing and end‐uses is traceable to the early 70s.^[3]^ This effort is now being enhanced by the emergence of approaches to translate force‐accelerated backbone bond dissociations into mechanistically and kinetically diverse chemistries, which underlies the rapidly increasing R&D activity in polymer mechanochemistry in the last decade.

We suggest that the potential of polymer mechanochemistry is broader still, because its experimental, computational and conceptual tools can meaningfully accelerate the effort to gain atomistic, quantitative, predictive, exploitable and generalizable understanding of some of the most fundamental problems in soft‐matter physics. In this review we highlighted the most recent examples of polymer mechanochemistry being exploited to gain hitherto‐inaccessible molecular details into elastomer fracture, macromolecular rheology and the structure/activity relationship enabling kinetic allostery. For reasons of space we omitted a few slightly older analyses of mechanochemical experiments yielding mesoscale‐level insights into elastomer behavior[^53^, ^57^, ^66^, ^67^] and macromolecular rheology,^[68]^ and of mechanical non‐atomistic models of protein allostery.^[69]^

The studies highlighted above remain decidedly niche, which we attribute to the challenges and risks of research projects whose success is predicated on getting multiple distinct approaches (synthesis, measurements, quantum‐chemical computations and/or modeling) to converge on the same answer. Productive tactics require a careful selection of a measurable physical quantity to act as a quantitative molecularly‐interpretable reporter of the presumed key physical process, mechanophores to affect this quantity directly, and computational methods capable of bridging the disparate temporaspatial scales separating mechanochemical reactions and macroscopically observed behavior. The current paucity of reported applications of polymer mechanochemistry to studying complex physical processes at the molecular scale means that broad trends that can guide the design of such experiments are yet to emerge. We view these challenges as temporary, being neither fundamental nor practically unsurmountable.

## Conflict of interests

The authors declare no conflict of interest.

6

## Biographical Information

*Robert O'Neill obtained his MChem degree from Durham University for his work developing the synthesis of redox‐active cycloheptatrienes under the supervision of Prof. Paul McGonigal. He then completed a PhD at the University of Liverpool under the supervision of Prof. Roman Boulatov where he studied force responsive polymers as tools to probe broader questions in polymer science*.

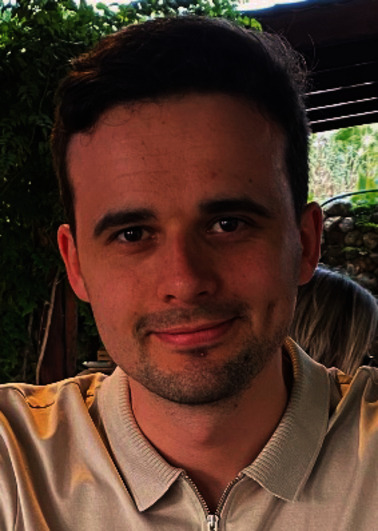

## Biographical Information

*Roman Boulatov earned a PhD at Stanford for work on metalloporphyrins, particularly for catalytic low‐temperature oxygen reduction under the supervision of Prof. James Collman. After a postdoc at Harvard with Prof. George Whitesides, where he explored unconventional means of energy conversion, he started his independent research program in polymer mechanochemistry at the UIUC before moving to Liverpool in 2012*.

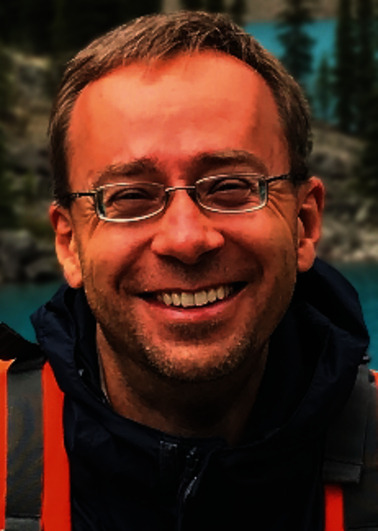

## Supporting information

As a service to our authors and readers, this journal provides supporting information supplied by the authors. Such materials are peer reviewed and may be re‐organized for online delivery, but are not copy‐edited or typeset. Technical support issues arising from supporting information (other than missing files) should be addressed to the authors.

Supporting Information

## Data Availability

The sources of the data graphed in Figure 1 are listed in Table S1
