# Supplementary material for: Mechanochemical Approaches to Fundamental Studies in Soft‐Matter Physics
Source: Angew Chem Int Ed Engl. 2024 Mar 15;63(19):e202402442. doi: 10.1002/anie.202402442 (PMC11497353; doi:10.1002/anie.202402442)
Supplement: Supplementary file 1 — Supporting Information [file ANIE-63-e202402442-s001.pdf]

Angewandte Chemie
Eine Zeitschrift der Gesellschaft Deutscher Chemiker

Supporting Information

Mechanochemical Approaches to Fundamental Studies in Soft-Matter Physics

*R. T. O'Neill, R. Boulatov**

Mechanochemical Approaches to Fundamental Studies in Soft-Matter Physics

Robert T. O'Neill and Roman Boulatov

Supporting Information

Table S1: sources and model chemistries of force-dependent activation free energies, $\Delta G^{\ddagger}$, plotted in Fig. 1C. In R2, R4, R6, R8-R11, the UHF designation is omitted for brevity. No $\Delta G^{\ddagger}$ data have been reported for R7. In all cases, the thermodynamic corrections were calculated in the pseudo-harmonic oscillator/rigid rotor/ideal gas approximation. Citation numbers refer to the bibliography of the mail text. See the cited references for values at different model chemistries and benchmarking analyses.

R1	B3LYP/6-311+G(d)	[38]
R2	CAM-B3LYP/6-311+G(d)/CPCM=DMSO	[16]
R3	CAM-B3LYP/6-311+G(d)	[34]
R4	BMK/6-31+G(d)	[61]
R5	B3LYP/6-311+G(d)/SMD=H2O	[8]
R6	MPW1K/6-31+G(d)	[5]
R8	MPW1K/6-31+G(d)	[32]
R9	BMK/6-31+G(d)	[30]
R10	MPW1K/6-31+G(d)	[13]
R11	CAM-B3LYP/6-311+G(d)	[5]
PS	BMK/6-31+G(d)	[61]
